# Sirolimus use in allogeneic hematopoietic cell transplant recipients: assessing its senotherapeutic role in a high risk population

**DOI:** 10.3389/fragi.2025.1673230

**Published:** 2025-10-14

**Authors:** Najla El Jurdi, Heba ElHusseini, Qing Cao, Thomas Klinger, Ella Shapiro, Melike Cömert, Mark Juckett, Shernan G. Holtan, Matthew J. Yousefzadeh

**Affiliations:** ^1^ Division of Hematology, Oncology, and Transplantation, Department of Medicine, University of Minnesota, Minneapolis, MN, United States; ^2^ Immune Deficiency Cellular Therapy Program, Center for Cancer Research, National Cancer Institute, National Institutes of Health, Bethesda, MD, United States; ^3^ Masonic Cancer Center, University of Minnesota, Minneapolis, MN, United States; ^4^ Columbia Center for Translational Immunology and Burch-Lodge Center for Human Longevity, Department of Medicine, Columbia University Medical Center, New York, NY, United States; ^5^ Roswell Park Comprehensive Cancer Center, Department of Medicine, Buffalo, NY, United States

**Keywords:** hematopoietic cell transplantation, cellular senescence, aging, cancer, sirolimus

## Abstract

Allogeneic hematopoietic cell transplantation (HCT) is often the only curative therapy for hematologic malignancies. Immune suppression is necessary for the engraftment of donor cells and prevention of graft-versus-host disease (GVHD). mTOR inhibitors like sirolimus are commonly used for GVHD prophylaxis. Low doses of sirolimus have demonstrated a gerotherapeutic effect, extending lifespan in animals, reducing senescent cell burden, and improving immune function in animals and humans. We hypothesized that the use of sirolimus in GVHD prophylaxis platforms, even at high doses, could have a senotherapeutic effect. We compared senescent cell burden in double umbilical cord blood HCT recipients with available baseline, day 100 and 365 post-HCT samples. All patients received an identical conditioning regimen with different GVHD prophylaxis: sirolimus + mycophenolate mofetil (MMF) or cyclosporine + MMF. At target doses to reduce GVHD risk, neither expression of senescence markers nor the abundance of SASP factors differed significantly in the sirolimus treated cohort compared to cyclosporine control cohort. However, we note a non-significant but perhaps biologically relevant trend of lower relative expression of *p16* ^*INK4a*^ and *p21* ^*CIP1*^ post-HCT in the sirolimus cohort. Further longitudinal analysis including a larger cohort would be useful to determine the true magnitude of differences in senescent cell burden. Our results suggest that the daily administration and dosing used for GVHD prevention are less likely to confer clinical benefits, possibly indicating that the beneficial effects of sirolimus occur within a specific therapeutic window. These findings highlight the need to further investigate senotherapeutic approaches in this setting of accelerated aging.

## Introduction

Allogeneic hematopoietic cell transplantation (HCT) is the only potentially curative therapy for hematological malignancies and other conditions. HCT comes at a cost of considerable morbidity and accelerated aging, cellular senescence and frailty as a result of exposure to high doses of chemotherapy and radiation leading to DNA damage and muscle loss equivalent to >20 years in the first few weeks of HCT (Jarden et al., 2009). Iatrogenic genotoxicity is known to be a strong inducer of cellular senescence, which can contribute to inflammaging and cause premature aging (Yousefzadeh M. et al., 2021). Both endogenous and exogenous forms of stress can induce cellular senescence, a cell fate whereby cells typically do longer proliferate but are still metabolically active (Shay and Wright, 2000; Zhang et al., 2022a). Senescent cells accumulate throughout the body (including lymphoid cells) with age and disease and are known to have a detrimental effect on both healthspan (the period of disease-free survival) and lifespan (Krishnamurthy et al., 2004; Baker et al., 2016; Yousefzadeh et al., 2020; Ogrodnik et al., 2017). Even though these cells lose their proliferative capacity, they are still metabolically active and resistant to apoptosis (Wiley and Campisi, 2021; Wang, 1995; Baar et al., 2017; Prieto et al., 2020; Childs et al., 2014). Senescent cells can take on a phenotype by which they secrete inflammatory chemokines, cytokines, growth factors, and matrix metalloproteases in what is known as the senescence-associated secretory phenotype (SASP) (Coppe et al., 2010). These SASP factors can act through autocrine, paracrine, or endocrine mechanisms interfering tissue homeostasis and contributing to the overall condition of increased inflammation during aging, even under non-pathogenic conditions, known as inflammaging (Zhang et al., 2022a; Franceschi and Campisi, 2014). Inflammaging is known to directly contribute to age-related decline in function and predispose individuals to frailty, disability, and chronic morbidity, enhancing the risk of premature death (Ferrucci and Fabbri, 2018). Senescent cell burden is often assessed by measuring expression of classical senescence makers *p16* ^*INK4a*^ and *p21* ^*CIP1*^, whose expression drives cell cycle arrest or by quantifying circulating SASP factors which can cause secondary senescence and disrupt tissue homeostasis (Coppe et al., 2010; Ogrodnik et al., 2024).

Inhibition of the mammalian target of rapamycin (mTOR) pathway with sirolimus (also known as rapamycin) is commonly used to prevent graft-versus-host disease (GVHD). Rapamycin and other rapalogs are potent gerotherapeutics, whose treatment have been shown to extend lifespan in multiple animal models, reduce markers of inflammation and senescent cell burden, improve immune function and age-related pathologies (Harrison et al., 2009; Mannick et al., 2014; Mannick et al., 2018; Yousefzadeh M. J. et al., 2021; Mannick and Lamming, 2023). From *in vitro* cell culture and animal model studies, rapamycin has been shown to be senomorphic, in that it does not selectively eliminate senescent cells like a senolytic but rather suppresses their inflammatory phenotype (Zhang et al., 2022a; Mannick and Lamming, 2023; Zhang et al., 2022b). Gerotherapeutic application of sirolimus using low-dose (below those used for GVHD prophylaxis) and intermittent administration was shown to be safe over a 48-week randomized clinical study in a healthy human cohort (Moel et al., 2025). However, it remains unknown whether the dose of sirolimus used to prevent GVHD is amenable to providing a gerotherapeutic benefit in HCT patients.

To date, no studies assessed the differential impact of sirolimus on aging biomarkers and HCT outcomes in humans. Therefore, we proposed investigating the role of sirolimus in mitigating accelerated aging in this high-risk population by assessing multiple aging mechanisms. We present the first in-depth examination of the potential senotherapeutic role of sirolimus in HCT recipients through the analysis of longitudinal pre- and post-HCT samples from two groups of umbilical cord blood (UCB) recipients undergoing a reduced intensity conditioning (RIC) transplantation with an identical conditioning regimen but with either sirolimus or calcineurin inhibitor (cyclosporine) based GVHD prophylaxis. Although the use of UCB has declined in recent years with the advent of new GVHD prophylaxis platforms allowing the use of haploidentical and mismatched unrelated donors, the importance and uniqueness of this pilot analysis and access to these samples lies in the use of UCB as the graft source to test our hypothesis. UCB provides a distinct advantage for studying the effects of sirolimus by eliminating the variability related to donor age and health status. This reduces potential confounding effects from graft source and donor age and characteristics in aging and senescence studies and strengthens the internal validity of our comparison between the cohorts with varying GVHD prophylaxis regimens.

## Methods

### Study design and inclusion criteria

**FIGURE 1:**
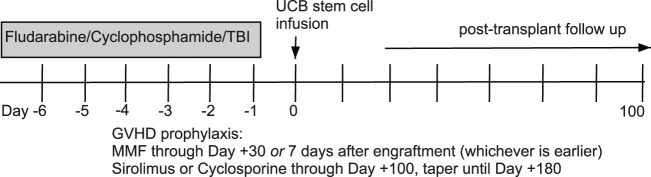
Allogeneic hematopoietic cell transplantation conditioning regimen and graft-versus-host disease prophylaxis.

This is a secondary analysis of bio banked samples from two prospective phase II clinical trials of RIC double UCB HCT at the University of Minnesota (Bejanyan et al., 2016). We identified all patients with existing pre-HCT and post-HCT day 100 and 1 year (day 365) samples from two UCB recipient groups undergoing HCT with an identical RIC regimen of fludarabine/cyclophosphamide/total body irradiation with either: (1) sirolimus + mycophenolate mofetil (MMF) or (2) cyclosporine + MMF as GVHD prophylaxis (Figure 1). The primary objective was to compare *p16* ^*INK4a*^ and *p21* ^*CIP1*^ aging biomarkers’ expression in the sirolimus (Siro) *versus* cyclosporine (CSA) based GVHD prophylaxis groups. The secondary objectives were to compare the expression of senescence-associated secretory phenotype (SASP) factors. All patients signed a written informed consent for clinical research. This study was reviewed and approved by the University of Minnesota Institutional Review Board. Supplemental clinical data was extracted from the electronic health record.

### Analysis of senescence marker expression in mononuclear cells

Total RNA was isolated from mononuclear cell pellets using the PureLink RNA Mini kit (Thermo Fisher). cDNA synthesis reactions were performed using 50 ng of total RNA with the High-Capacity cDNA Reverse Transcription Kit with RNase inhibitor (Thermo Fisher). 0.25 ng of cDNA was used to determine expression of *CDKN1A*/*p21* ^*CIP1*^, *CDKN2A*/*p16* ^*INK4a*^ and *18s* RNA by Taqman qPCR using a MIC-qPCR real time thermocycler (Bio Molecular Systems). Expression of *p16* ^*INK4a*^ and *p21* ^*CIP1*^ was normalized by determination of ∆Ct^-1^ (for individual data points) or ∆∆Ct relative quantification (for longitudinal analysis where expression was normalized to the baseline timepoint). All qPCR reactions were run in triplicate. Probe information for as described in: *18S* (Taqman ID Hs03003631_g1); *p21* ^*CIP1*^ (Taqman ID Hs00355782_m1) *p16* ^*INK4a*^ (Taqman Custom Assay ID APWCZMM) (El Jurdi et al., 2024).

### Analysis of circulating senescence-associated secretory phenotype factors

Multiplexed ELISA was performed to quantify circulating SASP factors in serum specimens using the Human Cytokine Proinflammatory Focused 15-Plex Discovery Assay (GM-CSF, IFNɣ, IL-1β, IL-1RA, IL-2, IL-4, IL-5, IL-6, IL-8, IL-10, IL-12p40, IL12-p70, IL-13, MCP-1, TNF⍺) and Human Cardiovascular Disease Panel 2 (GDF15) multiplex kits (Millipore/Eve Technologies) on the Luminex 100 system (Luminex) system with each sample being run in duplicate (Miller et al., 2025). In cases where the analyte was below the limit of detection in a sample, half the value of the lowest measured value for that analyte was assigned for the purposes of analysis.

### Statistical methods

Descriptive statistics, including median and range, were employed to summarize demographic, laboratory, and clinical continuous variables across the two groups (Siro vs. CSA). Categorical variables were summarized using frequency counts and percentages with confidence intervals (CIs). Comparisons between groups were conducted using Wilcoxon rank-sum tests for continuous variables and chi-square test or Fisher’s exact test in cases of limited expected counts for categorical variables. Quantile regression at the median was planned to adjust for sex or BMI if significant in univariate analyses; however, adjustment was not required as no significant differences based upon sex or BMI were observed. Overall survival (OS) and progression free survival (PFS) were estimated using Kaplan-Meier, with differences in curves estimated by log-rank tests (Kaplan et al., 1992). The cumulative incidence function was used to calculate probabilities and 95% CI of treatment-related mortality (TRM), relapse, and acute GVHD (Lin, 1997). For relapse and acute GVHD, death without the event was the competing risk, while for TRM, the competing event was relapse. Fine and Gray regression was applied to compare estimates between groups (Fine and Gray, 1999).

All statistical tests were two-sided, and significance was established at p < 0.05. The statistical analyses were conducted using SAS 9.4 (SAS Institute, Inc., Cary, NC) and R version 4.2.2 (R Foundation for Statistical Computing, Vienna, Austria).

## Results

### Patient characteristics

Patients were enrolled on the primary transplant protocols between October 2010 and November 2016. A total of 53 patients were included in this secondary analysis, 22 in the cyclosporine (CSA) cohort and 31 in the sirolimus (Siro) cohort. Table 1 shows patient, disease, and transplant characteristics. There were no significant differences in baseline characteristics between the two cohorts. Median age at HCT was 60 years (min-max, 24-73) overall, 58 (24-69) for the CSA cohort and 62 (27-73) for the Siro cohort. There were more male receipts in both groups, 59% in the CSA cohort and 74% in the Siro cohort. There was more female donor to male recipients in the Siro cohort, 58% *versus* 41%. There was so significant difference between the two cohorts in HCT outcomes including treatment related mortality, overall and progression free survival, relapse, and acute or chronic GVHD; therefore, aging biomarkers were not assessed for correlation with HCT specific outcomes.

**TABLE 1:** Patient, disease, and transplant characteristics.

	All Groups	Cyclosporine	Sirolimus
Patients (N)	53	22	31
Age at HCT			
Median (Min-Max)	60 (24–73)	58 (24–69)	62 (27–73)
Sex			
Male	36 (68%)	13 (59%)	23 (74%)
Female	17 (32%)	9 (41%)	8 (26%)
Diagnosis			
ALL	8 (15%)	2 (9%)	6 (19%)
AML	18 (34%)	9 (41%)	9 (29%)
CLL	3 (6%)	2 (9%)	1 (3%)
MDS	7 (13%)	4 (18%)	3 (10%)
Myeloma	3 (6%)	0	3 (10%)
NHL/HL/other	14 (26%)	5 (23%)	9 (29%)
Body Mass Index			
Median (Min-Max)	27 (19–42)	28 (19–42)	27 (21–40)
Donor to Recipient Sex			
Female to Male	27 (51%)	9 (41%)	18 (58%)
Other	26 (49%)	13 (59%)	13 (42%)
HLA-matching			
4/6	19 (36%)	4 (18%)	15 (48%)
5/6	26 (49%)	14 (64%)	12 (39%)
6/6	7 (13%)	4 (18%)	3 (10%)
Missing	1 (2%)	0	1 (3%)
Recipient CMV Status			
Positive	24 (45%)	13 (59%)	11 (36%)
Negative	29 (55%)	9 (41%)	20 (64%)

Abbreviations: HCT, hematopoietic cell transplantation; ALL, acute lymphocytic leukemia; AML, acute myeloid leukemia; CLL, chronic lymphocytic leukemia; MDS, myelodysplastic syndrome; NHL, Non-Hodgkin Lymphoma; HL, hodgkin lymphoma; CMV, cytomegalovirus.

### Comparison of *p16* ^*INK4a*^ and *p21* ^*CIP1*^ expression, surrogate biomarkers of aging, in PBMCs

**FIGURE 2:**
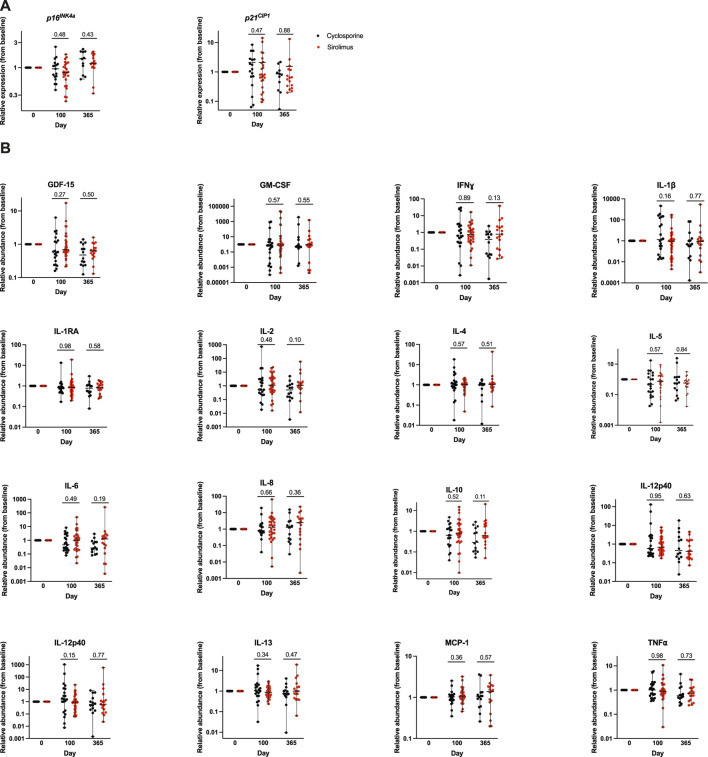
Relative expression of senescence marker and circulating SASP factors compared to baseline in each patient at each subsequent post-HCT timepoints, day 100 and 1 year. **(A)** Whole blood from each of the patient cohorts receiving either cyclosporine based or sirolimus based graft-versus-host disease prophylaxis was used to isolate peripheral blood mononuclear cells by magnetic bead purification. Total RNA was used to quantify expression of the cellular senescence marker *p16* ^*INK4a*^ and *p21* ^*CIP1*^ by qPCR. Expression was normalized to *18s*. Data is represented in box and **(B)** Relative abundance of SASP factors (to baseline) were quantified in plasma samples by multiplexed ELISA. Values represent the median and the range.

We assessed expression of the senescence markers *p16* ^*INK4a*^ and *p21* ^*CIP1*^ *,* in peripheral blood mononuclear cells (PBMCs), a recognized surrogate biomarker for biological aging that can be applied to human samples (El Jurdi et al., 2024; Miller et al., 2025; Liu et al., 2011; Liu et al., 2009; Englund et al., 2021; Sanoff et al., 2014). Baseline *p16* ^*INK4a*^ expression (∆Ct^-1^ *p16* ^*INK4a*^) was similar in both groups with median of 0.100 (min-max 0.091–0.117) and 0.102 (0.092–0.123) in the CSA and Siro cohorts, respectively. We then looked at the relative expression of *p16* ^*INK4a*^ and *p21* ^*CIP1*^ compared to baseline in every patient at each subsequent post-HCT timepoints, day 100 and 1 year (Figure 2A). At day 100 post-HCT, median relative expression of *p16* ^*INK4a*^ was 1.0 (0.4–2.5) in the CSA cohort similar to baseline, and 0.8 (0.2–1.8) in the Siro cohort, indicating a non-significant trend to lower levels compared to baseline (p = 0.48, comparing levels in both cohorts). At 1-year post-HCT, median relative expression was 1.5 (0.6–2.3) in the CSA group and 1.2 (0.3–2.0), both increased relative to baseline expression, with a non-significant trend for higher expression in the CSA cohort (p = 0.43). Baseline *p21* ^*CIP1*^ expression (∆Ct^-1^ *p21* ^*CIP1*^) was similar in both groups with median of 0.064 (0.054–0.079) and 0.065 (0.057–0.078), and in the CSA and Siro cohorts, respectively. Day 100 *p21* ^*CIP1*^ median relative expression was 1.2 (0.1–8.4) in the CSA cohort and 0.8 (0.1–14.2) in the Siro cohort (p = 0.47), while 1-year relative expression was 0.8 (0.1–2.2) in the CSA cohort and 0.6 (0.2–13.2) in the Siro cohort, indicating a non-significant trend for lower expression compared to baseline (p = 0.88).

### Comparison of expression of senescence-associated secretory phenotype (SASP) factors

We quantified the levels of 15 SASP factors (GM-CSF, IFNɣ, IL-1β, IL-1RA, IL-2, IL-4, IL-5, IL-6, IL-8, IL-10, IL-12p40, IL12-p70, MCP-1, TNF⍺, and GDF15). Baseline, day 100, and 1 year post-HCT SASP levels were not significantly different between the two cohorts. We then looked at the relative abundance of each SASP factor compared to baseline in every patient at each subsequent post-HCT timepoints (Figure 2B). There was no statistically significant difference in the relative expression of any of the SASP factors at subsequent post-HCT assessment timepoints. However, at day 100 we note a trend for higher relative abundance of IL-1β (p = 0.16), and IL-12p70 (p = 0.15) in the CSA cohort; and at 1 year we note a trend for lower IL-2 (p = 0.10), IL-6 (p = 0.19), and IL-10 (p = 0.11) abundance in the CSA cohort.

## Discussion

We present the first pilot in-depth examination of the potential senotherapeutic role of sirolimus in HCT recipients through the analysis of longitudinal pre- and post-HCT samples from two groups of UCB recipients undergoing a reduced intensity conditioning HCT with an identical conditioning regimen. Comparison of patients treated with Siro *versus* CSA to prevent GVHD did not reveal statistically significant differences in senescence marker expression in PBMCs or in circulating SASP abundance. Our results suggest that the daily administration and dosing used for GVHD prevention are less likely to confer benefits in the context of aging, possibly indicating that the senotherapeutic and broad gerotherapeutic effects of sirolimus likely occur within a specific therapeutic window. However, we note a non-significant but perhaps biologically relevant trend of lower median relative expression of *p16* ^*INK4a*^ and *p21* ^*CIP1*^ at the post-HCT timepoints in the Siro cohort compared to the CSA cohort. Further longitudinal analysis including a larger cohort would be useful to determine the true magnitude of differences in markers of senescent cell burden and to assess whether changes may emerge later on, if at all. Additionally, a dose-escalation study would be important to determine the upper limit of sirolimus treatment that maintains beneficial age-related effects.

Analysis of senescence in newly repopulated T cells that arise from the UCB donor may not be representative of the level of senescent cell burden in the solid organs of the transplant recipients that have undergone TBI and chemotherapy (Englund et al., 2021; Wang et al., 2020; Demaria et al., 2017; Shaf et al., 2022). Therefore, future studies would benefit from measuring senescent cell burden in solid tissue biopsies from accessible parenchymal tissue (i.e., adipose, muscle, or skin) to complement assessment of senescence marker expression in newly developed immune cells across different time points. This methodology could also help determine the sensitivity of donor immune cells to secondary senescence originating from the parenchyma. Furthermore, future investigations should, when possible, include the evaluation of donor senescence markers.

We had a unique opportunity to investigate a research hypothesis with high potential impact, targeting a high-risk population that stands to benefit the most from future interventional studies, potentially extending beyond the transplant setting. First, our comparative groups underwent HCT from the same donor source using an identical multi-agent conditioning regimen (chemotherapy + radiation), with GVHD prophylaxis as the only variable in the treatment intervention. Second, the use of UCB donor source offers a distinctive advantage in studying the effect of sirolimus, as it eliminates the potential confounding effects of donor source and donor age on the subsequently observed aging phenotype. The importance and uniqueness of this pilot analysis and access to these samples lies in the use of UCB as the graft source to test our hypothesis and strengthens the internal validity of our comparison between the cohorts with varying GVHD prophylaxis regimens. However, our retrospective analysis was limited by a smaller sample size, heterogenous population of recipients with different underlying hematological malignancies, as well as the use of archival samples. Initiating prospective studies that incorporate longitudinal measurements of healthspan, and biomarkers of aging could better elucidate how HCT influences aging and whether the use of mTOR inhibitors for GVHD prophylaxis may confer gerotherapeutic benefits, depending on the dosing strategy. Incorporation of biomarkers of aging into future studies could provide the opportunity to see if these endpoints have predictive power with respect to the onset of GVHD or correlate with its severity. Furthermore, more robust analyses of the hallmarks of aging may provide insights into additional gerotherapeutic approaches to mitigate the age-accelerating effects of both the underlying condition and its treatment, conditioning regimen for HCT, donor and graft source, GVHD prophylaxis, as well as post-HCT complications, each of which may uniquely impact individual patients. Our findings from this pilot analysis highlight the need for further investigation of senotherapeutic approaches in this clinical setting of accelerated aging.

## Data Availability

The raw data supporting the conclusions of this article will be made available by the authors, without undue reservation.
